# The Pyridoxal-5′-Phosphate-Dependent Enzymes of *Mycobacterium tuberculosis*

**DOI:** 10.1021/acsinfecdis.5c00996

**Published:** 2026-01-30

**Authors:** Alessio Peracchi, Bienyameen Baker

**Affiliations:** † Department of Chemistry, Life Sciences and Environmental Sustainability, University of Parma, Parma I-43124, Italy; ‡ SAMRC Centre for Tuberculosis Research; Division of Molecular Biology and Human Genetics, Faculty of Medicine and Health Sciences, Stellenbosch University, Cape Town 7505, South Africa

**Keywords:** *Mycobacterium tuberculosis*, PLPome, functional annotation, sulfur metabolism, essential enzymes, drug targets

## Abstract

Enzymes that depend on the cofactor pyridoxal 5′-phosphate (PLP) catalyze a remarkable variety of biochemical reactions in all organisms. In particular, the genome of *Mycobacterium tuberculosis*, the causative agent of tuberculosis (TB), encodes 45 bona fide PLP-dependent enzymes plus a few related proteins that presumably do not have enzymic function. The large majority of the 45 enzymes have been characterized in terms of catalytic activity and structure. Several of them have been shown to be central to the bacterium’s survival and pathogenicity, while some of these enzymes are targets of an extant drug (d-cycloserine). Herein, the annotated catalog of the PLP-dependent enzymes in *M. tuberculosis* is presented and analyzed with three main goals in mind. The first will be to assess the specific aspects of mycobacterial metabolism that rely most on PLP-dependent enzymes. A second goal will be to signal those enzymes whose function is still uncertain and whose functional characterization may help to further understand the biology of *M. tuberculosis*. Finally, we will examine the potential and limitations of targeting the PLP-dependent enzymes for the development of new antimycobacterial drugs.

## Graphical abstract

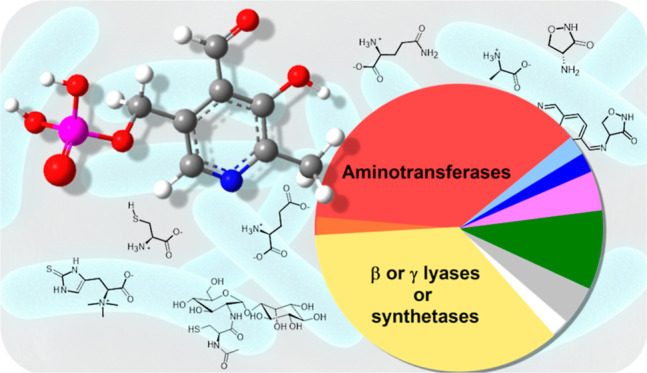

## Introduction

Pyridoxal 5′-phosphate (PLP) is the biologically active form of vitamin B6.^1^ PLP-dependent enzymes occur in all living organisms and are indispensable in the biosynthesis and degradation of amino compounds,^2,3^ including many belonging to central metabolism and others that are secondary metabolites.^4–6^

PLP-dependent enzymes exhibit remarkable functional diversity: as of 2025, the Enzyme Commission (E.C.) registers more than 230 PLP-dependent catalytic functions (https://bioinformatics.unipr.it/cgi-bin/bioinformatics/B6db/home.pl).^3^ The role of PLP-dependent enzymes is pivotal in particular for the metabolism of amino compounds, where these catalysts carry out (among others) a wide array of transamination, decarboxylation, racemization, and elimination reactions.^2,7^ Such catalytic versatility is largely due to the reactive nature of the PLP cofactor.^7,8^ Upon binding of the substrate to the enzyme, PLP typically reacts with the α-amino group of the substrate, forming a Schiff base; this covalent linkage allows the stabilization of different carbanionic intermediates that arise during the reaction cycle^7,9,10^ (Figure 1).

**Figure 1:**
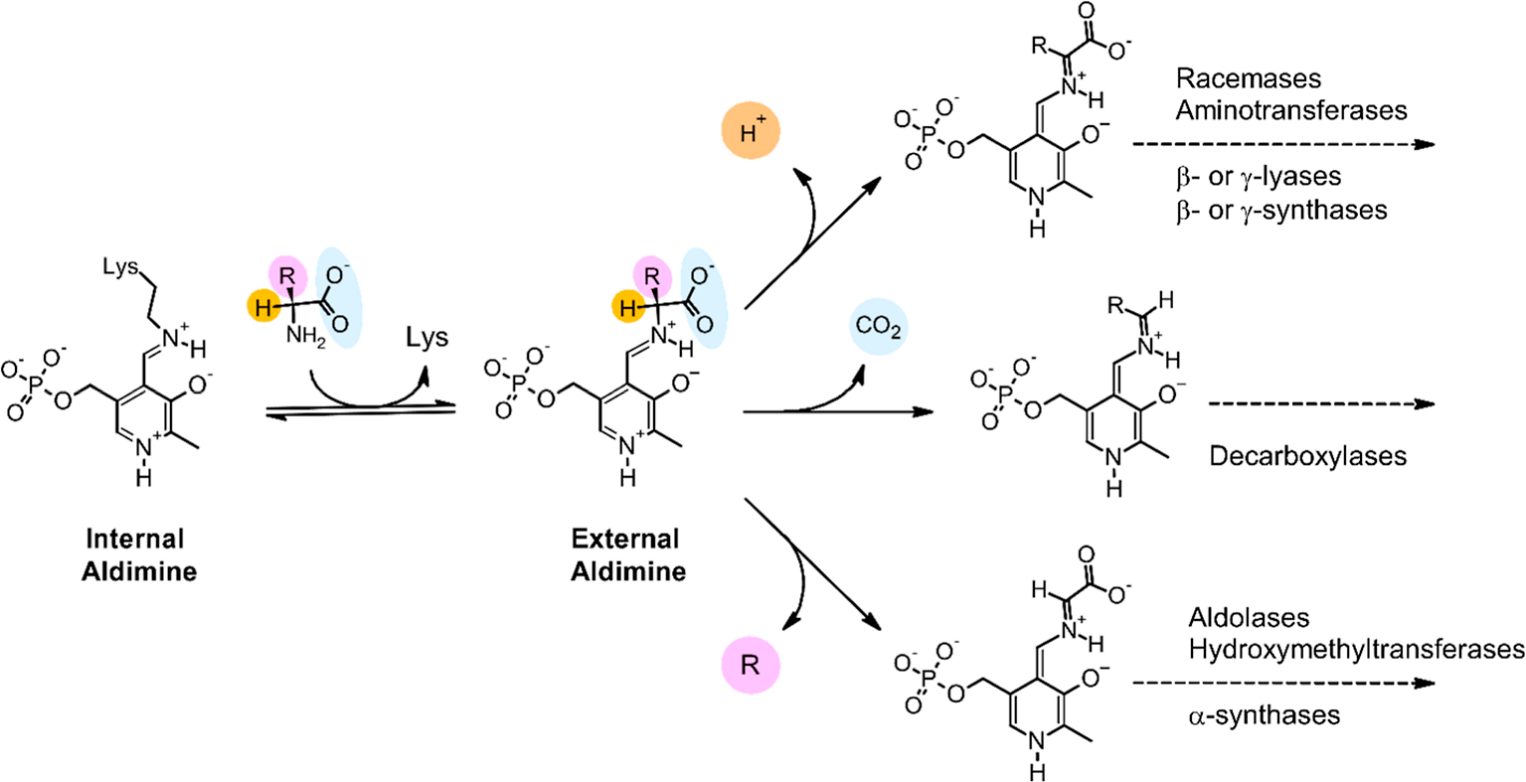
Most common reaction types catalyzed by PLP-dependent enzymes that act on amino acids. In these enzymes, the cofactor is typically bound to the ε-amino group of a lysine residue, forming a Schiff base (internal aldimine). Binding of a substrate leads to the formation of an external aldimine intermediate, with release of the lysine side chain. Subsequently, the protonated ring system of PLP acts as an electron sink, to stabilize species carrying a negative charge on the α-carbon (carbanions). Depending on the specific enzyme, such stabilized carbanions (termed quinonoids) can be formed upon cleavage of any of the three covalent bonds connecting the α-carbon to its substituents, as shown in the rightmost part of the figure.

Despite the variety of catalytic activities they display, PLP-dependent enzymes belong to a small number of independent evolutionary lineages and fall into just seven structural classes, called “fold types” and labeled with Roman numerals (I to VII).^3,11^ Among these, fold type I is the most frequently encountered, as it encompasses the vast majority of documented transaminases but also a range of other enzymes with diverse activities.

Tuberculosis (TB) is a contagious disease, caused by the pathogen *Mycobacterium tuberculosis* (*Mtb*), which represents a huge burden to human health. According to the latest WHO’s Global Tuberculosis Report,^12^ around 8.2 million people were newly diagnosed with active TB in 2023 and 1.25 million people worldwide died from the disease. These high numbers and the emergence of antibacterial-resistant strains underscore the importance of identifying new drugs and drug targets in *Mtb*. Like most bacteria (as well as Archea, plants, and fungi), *Mtb* synthesizes PLP through a two-enzyme pathway^13^ (Supporting Information, Figure S1) and requires the cofactor for various metabolic processes. It has been shown that vitamin B6 is essential in order for *Mtb* to survive and remain virulent, implying that enzymes dependent on this cofactor play crucial roles in mycobacterial processes.^13^

In this review, we first present a list of the PLP-dependent enzymes encoded in the *Mtb* genome,^14^ which, to our understanding, represents the complete set (i.e., the “complement”) of these enzymes in this pathogen. Such an annotated catalog helps appreciate how much research has been carried out on these enzymes and provides a peculiar perspective on several aspects of *Mtb* biology. Furthermore, this catalog pinpoints those enzymes that still remain to be classified in terms of function. Finally, as d-cycloserine (DCS—an inhibitor of PLP-dependent enzymes and of alanine racemase in particular) has been historically used as an antitubercular drug, it is possible that PLP-dependent enzymes could include other plausible targets for drug development. We will hence evaluate which enzymes represent the most promising targets for possible new antimycobacterial drugs, summarizing also the drug discovery efforts that may have been done toward such targets. Throughout this analysis, selected PLP-dependent enzymes will be examined in detail, focusing on their role in *Mtb* metabolism and their druggability.

### Assembling a Census of the PLP-Dependent Enzymes in *M. tuberculosis*

An initial inventory of the PLP-dependent enzymes encoded in the *Mtb* H37Rv strain genome^14^ was retrieved from the B6 database (https://bioinformatics.unipr.it/cgi-bin/bioinformatics/B6db/home.pl).^3^ The list was checked and integrated through a text search in the NCBI website (https://www.ncbi.nlm.nih.gov/protein), using “pyridoxal” as a keyword in the organism “*Mycobacterium tuberculosis* H37Rv” and excluding results from the PDB database (sequences derived from structural studies). Initially this search yielded 87 hits, many of which, however, were not pertinent (such as enzymes involved in the biosynthesis of PLP) or represented duplicate entries or pseudogenes. Eventually, the results of these searches converged on a set of 45 gene products that is summarized in Table 1 and presented in a more extended form in Supporting Information, Table S1.

**Table 1:** PLP-Dependent Enzymes Encoded in the *Mtb* H37Rv Genome^a^

Rv locus	gene name	Established or putative catalytic function	EC#	Validation	Ref	PDB	Essential?
Rv0032	*bioF2*	Serine C-palmitoyltransferase	2.3.1.50 ?	-	-	-	1/4
Rv0070c	*glyA2*	Serine hydroxymethyltransferase	2.1.2.1	B	^20^	-	0/4
Rv0075	*-*	L-cysteine desulfidase (C-S lyase)	4.4.1.28 ?	-	-	-	0/4
Rv0337c	*aspC*	Alanine aminotransferase	2.6.1.2	B	^21^	-	3/4
Rv0391	*metZ*	O-succinylhomoserine sulfhydrylase	2.5.1.-	G	^22^	3NDN	1/4
Rv0524	*hemL*	Glutamate-1-semialdehyde 2,1-aminomutase	5.4.3.8	-	-	-	4/4
Rv0812	*pabC*	4-amino-4-deoxychorismate lyase / D-amino acid transaminase	4.1.3.38/2.6.1.21	B	^23^	6Q1Q	1/3
Rv0848	*cysK2*	S-sulfo-L-cysteine synthase	2.8.5.1	B	^24^	-	0/4
Rv0858c	*dapC*	Glutamine transaminase^b^	2.6.1.64/117	-	-	2O0R	0/4
Rv0884c	*serC*	Phosphoserine aminotransferase	2.6.1.52	B	^25^	2FYF	3/3
Rv1077	*cysM2*	Cystathionine beta-synthase	4.2.1.22	B	^26^	7XNZ	0/4
Rv1079	*metB*	Cystathionine gamma-lyase	2.1.2.1	B	^27,28^	-	0/3
Rv1093	*glyA*	Serine hydroxymethyltransferase	4.4.1.1	B	^20^	6ULD	3/3
Rv1178	*-*	Succinyldiaminopimelate transaminase	2.6.1.17	B	^29^	-	0/4
Rv1293	*lysA*	Diaminopimelate decarboxylase	4.1.1.20	B	^30,31^	1HKV	4/4
Rv1295	*thrC*	Threonine synthase	4.2.3.1	B	^32^	2D1F	4/4
Rv1328	*glgP*	Glycogen (glucan) phosphorylase	2.4.1.1	-	-	-	0/4
Rv1336	*cysM*	[CysO]-thiocarboxylate-dependent L-Cys synthase	2.5.1.113	B	^33,34^	3DWG	0/4
Rv1464	*csd*	Cysteine desulfurase (SufS-type)^c^	2.8.1.7	B	^35^	8ODQ	4/4
Rv1559	*ilvA*	Threonine ammonia-lyase	4.3.1.19	B	^36^	-	2/3
Rv1568	*bioA*	S-adenosylmethionine : 8-amino-7-oxononanoate aminotransferase	2.6.1.62	B	^37^	3BV0	1/4
Rv1569	*bioF1*	8-amino-7-oxononanoate synthase	2.3.1.47	B	^38^	-	0/3
Rv1600	*hisC*	Histidinol-phosphate aminotransferase	2.6.1.9	B	^39^	4R8D	4/4
Rv1612	*trpB*	Tryptophan synthase beta subunit	4.2.1.20	B	^40^	5OCW	3/3
Rv1655	*argD*	Acetylornithine aminotransferase	2.6.1.11	B	^41^	7NN1	4/4
Rv1832	*gcvB*	Glycine dehydrogenase (decarboxylating)	1.4.4.2	-	-	-	3/4
Rv2148c	*-*	PLP-homeostasis protein (YggS family)	?	-	-	-	0/3
Rv2210c	*ilvE*	Branched-chain amino acid transaminase	2.6.1.42	B	^42^	3HT5	3/4
Rv2231c	*cobC*	Histidinol phosphate aminotransferase^d^	2.6.1.9 ?	B	^43^	-	1/3
Rv2294	*-*	Cystathionine β-lyase / L-cysteine desulfidase	4.4.1.8/28 ?	-	-	-	0/4
Rv2334	*cysK1*	O-acetylserine sulfhydrylase	2.5.1.47	B	^44^	2Q3B	0/3
Rv2531c	*-*	Putative amino acid decarboxylase	4.1.1.- ?	-	-	9N0O	0/4
Rv2589	*gabT*	4-aminobutyrate aminotransferase	2.6.2.19	-	-	-	0/4
Rv3025c	*iscS*	Cysteine desulfurase (NifS-type)	2.8.1.7	B	^45^	4ISY	3/4
Rv3290c	*lat*	L-lysine 6-transaminase	2.6.1.36	B	^46,47^	2CIN	0/4
Rv3329	*-*	Taurine:pyruvate aminotransferase	2.6.1.77 ?	-	-	-	0/4
Rv3340	*metC*	O-acetylhomoserine sulfhydrylase	2.5.1.49	G	^48^	-	0/3
Rv3402c	*-*	dTDP-4-amino-4,6-dideoxy-D-glucose aminotransferase	2.6.1.33	B	^49^	-	0/4
Rv3423c	*alr*	Alanine racemase	5.1.1.1	B	^50^	1XFC	4/4
Rv3432c	*gadB*	Glutamate decarboxylase	4.1.1.15	B	^51^	-	0/4
Rv3565	*aspB*	Valine:pyruvate aminotransferase	2.6.1.66	B	^21^	5YHV	1/2
Rv3684	*-*	L-cysteine desulfidase (C-S lyase)	4.4.1.28	B	^52^	-	0/4
Rv3700c	*egtE -*	Hercynylcysteine S-oxide lyase	4.4.1.36	G	^53^	-	0/3
Rv3722c	*-*	Aspartate aminotransferase	2.6.1.1	B	^21^	5C6U	4/4
Rv3772	*hisC2*	Aromatic amino acid transaminase	2.6.1.57	B	^39^	4R2N	0/4

a The reported catalytic function is based on experimental studies (when available) or on sequence similarity to functionally validated enzymes. Experimental validation could be based on either biochemical (B) or genetic (G) data—such as complementation or deletion studies. Question marks pinpoint gene products whose exact catalytic activity is most uncertain (see main text). Essentiality is based on the transposon mutagenesis studies of Sassetti et al.,^54^ Griffin et al.,^55^ DeJesus et al.,^56^ and Minato et al.,^57^ as summarized in the MycoBrowser website;^17^ the entry indicates the number of studies in which the gene was found essential, divided by the number of studies in which it was tested. E.g., 1/3 signals a gene that was found essential in one study out of three in which it was analyzed.

b The Rv0858c gene product is annotated and often referred to as “succinyldiaminopimelate aminotransferase” (DapC).^58^ Nonetheless, no experimental evidence has been reported to support the proposed activity, while the sequence of Rv0858c is much more closely related to aminotransferases acting on glutamine and methionine^59,60^ than to authentic DapC proteins. The function of Rv0858c might hence approximate that of human glutamine transaminase K.^61^ In contrast, the role of succinyldiaminopimelate aminotransferase in *Mtb* appears to be played by Rv1178,^29^ which is also 61% identical to the functionally validated succinyldiaminopimelate transaminase from *Corynebacterium glutamicum*.^62^

c Rv1464 belongs to a subgroup of enzymes that are reportedly active on both cysteine and selenocysteine (to produce elemental sulfur and selenium, respectively).^63^ The study that tested the biochemical function of Rv1464 only assessed its activity as a desulfurase.^35^

d Although Rv2231c has been recently shown to possess some histidinol phosphate aminotransferase activity,^43^ there are reasons to suspect such an activity does not mirror the actual biological function of the enzyme. First, based on the published data, the activity of Rv2231c was 2 or 3 orders of magnitude lower than the activity of Rv1600 (HisC) in the same reaction.^39^ Second, the protein was reportedly secreted and hence badly suited to contribute to the bacterial central metabolism. Third, while sequence similarity to validated histidinol phosphate aminotransferases is very weak, substrates other than histidinol phosphate were not tested. Finally, the protein had some impact in the interaction of *Mtb* with its host,^43^ suggesting that Rv2231c may be moonlighting and playing some important, possibly noncatalytic, role.

This list of gene products represents, to the best of our knowledge, the complement of bona fide PLP-dependent enzymes in *Mtb*—or the bacterium PLPome, according to a nomenclature put forward by some authors.^15,16^ Nonetheless, as the list is based ultimately on literature data and sequence similarity criteria, it is formally possible that some mycobacterial PLP-dependent enzyme(s) may be missing for at least two reasons. First, we cannot rigorously exclude that some as-yet undescribed PLP-dependent enzymes may not fall into the seven “fold-type” categories and therefore escape detection[Fn fn1]. Second, it is also theoretically possible that some PLP-dependent enzymes, structurally belonging to one of the known “fold-types”, may nevertheless have diverged so much from their congeners, as to become unrecognizable sequence-wise.

Information about each of the 45 sequences in Table 1 was initially retrieved through specialized databases such as Mycobrowser (https://mycobrowser.epfl.ch/)^17^ and TB database (http://tbdb.bu.edu/tbdb_sysbio/MultiHome.html).^18^ Literature concerning the individual sequences was mined using Paperblast^19^ and by text searches in Google Scholar. When the function of a given enzyme had not been established experimentally, its sequence was used for BLASTP analyses against the UniProt/Swissprot database to identify the closest homologous enzymes of known function. The existence of experimental 3D structures for the enzymes was assessed by BLAST searches in the Protein Data Bank (https://www.rcsb.org/).

### A Comparative Assessment of the *M. tuberculosis* PLPome

While Table 1 refers, as stated, to gene products from the laboratory strain H37Rv, a BLAST search toward the genomes of three other *Mtb* strains (F11, cdc 1551, and Haarlem) performed at the Integrated Microbial Genomes and Microbiomes website (https://img.jgi.doe.gov/cgi-bin/mer/main.cgi) indicated that all of the genes were very conserved in the *M. tuberculosis* complex. Furthermore, only seven of the genes in Table 1 lacked clear orthologs from the genome of *Mycolicibacterium smegmatis*, which is commonly used as a model organism to study mycobacteria (Supporting Information, Table S1; conversely, the genome of *M. smegmatis* encodes some PLP-dependent enzymes without clear orthologs in *Mtb*, data not shown). This is broadly consistent with the notion that, despite having evolved into an obligate pathogen, *Mtb* has retained a large proportion of the metabolic pathways found in its environmental, nonpathogenic ancestors, adapting and even increasing its metabolic repertoire to favor survival and propagation within the human host.^64^

The number of PLP-dependent enzymes in *Mtb* is not too far from the 61 enzymes estimated in *Bacillus subtilis* ^16^ and from the 53 enzymes (plus three related proteins with nonenzymic function) estimated in humans.^3^ However, despite comparable absolute numbers, different organisms may possess very different assortments of enzymes owing to different metabolic setups. In particular, while *Mtb* and *Homo sapiens* appear to possess similar overall numbers of PLP-dependent enzymes, less than one-half of the PLP-dependent activities in these organisms overlap (Supporting Information, Table S1).

### Distribution of Functions within the *M. tuberculosis* PLPome

As can be gleaned from Table 1, for the vast majority of the PLP-dependent gene products, the precise catalytic function has been established experimentally (through biochemical or genetic approaches), attesting to the intense research that has been conducted on the *Mtb* biochemistry. For a few other enzymes, activity can be inferred with good confidence based on a close homology to functionally validated enzymes. It should be stressed that, for several gene products in Table 1, the established or likely function is different from the annotations retrievable from public databases, in agreement with the well-known limits of such annotations.^65^

Most of the proteins in Table 1 are enzymes of primary metabolism, the majority of which are involved in the anabolism and catabolism of amino acids. Notably, *Mtb* does not possess clear homologues of enzymes involved in cyclization reactions or in reactions with oxygen, which are typically found in secondary metabolism.^4,5^ It is also noteworthy that several of the enzymes have multiple functions: the most striking example is Rv0812 (PabC), which has been shown to function both in the synthesis of folate and in the metabolism of d-amino acids.^23^ Rv1600 (HisC) and Rv3772 (HisC2) are transaminases possessing distinct and yet partially overlapping substrate specificities.^39^ There are also apparently duplicated functions, such as those of Rv1093 (glyA) and Rv0070c (glyA2): both are validated as serine hydroxymethyltransferases and show similar biochemical and kinetic properties,^20^ but only Rv1093 is essential for growth. In these instances, the apparently isofunctional enzymes may have, in fact, distinct functions in the cell or may be expressed under different conditions.

The largest group of enzymes is represented by transaminases (aminotransferases), which participate in the synthesis and breakdown of most amino acids and are crucial for maintaining the nitrogen balance within the cell (Figure 2). A body of evidence supports the notion that nitrogen metabolism is an essential determinant for the pathogenicity of *M. tuberculosis* and related species.^66–68^ Nitrogen metabolism affects the survival and virulence of *Mtb* in the harsh environment found in macrophages, characterized by low pH and low oxygen. Nitrogen metabolism is closely related to the dormancy state and drug resistance of the mycobacterium, which can use a variety of inorganic or organic nitrogen sources, including ammonium salts, nitrate, glutamine, and asparagine.^68^

**Figure 2:**
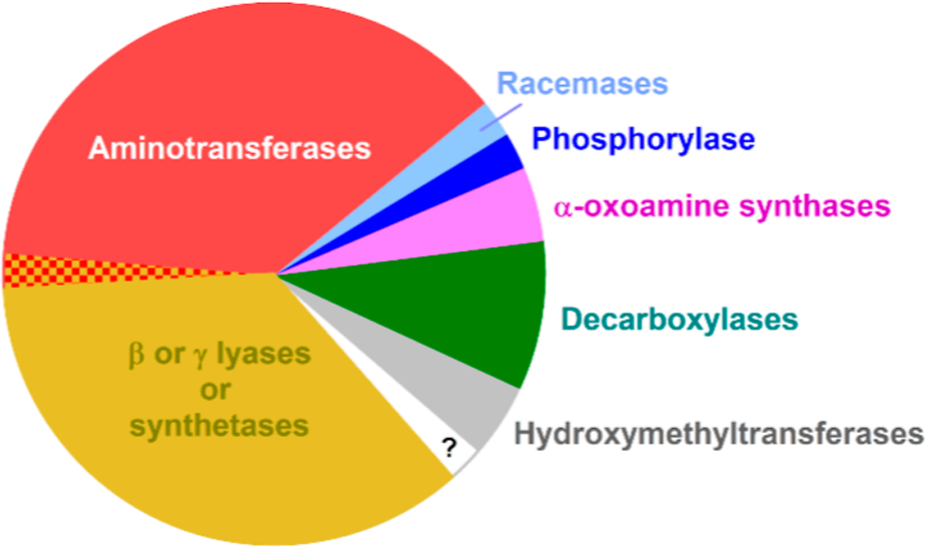
An overview of the functions of PLP-dependent enzymes in *Mtb*. The figure summarizes the data from Table 1. The aminotransferases sector includes Rv0524 (glutamate semialdehyde aminomutase) as its reaction encompasses an intramolecular amino group transfer.^69^ The checkered (red and gold) sector refers to Rv0812, an enzyme reported to possess both lyase and aminotransferase activities.^23^

Even though, as noted, there is typically some overlap in the specificity of transaminases acting on amino acids, the lack of some *Mtb* aminotransferases (such as Rv0884c, Rv1600, and Rv3722c) cannot be compensated by others for growth in vitro (Table 1). Beyond amino acid metabolism, transaminases are involved in the synthesis of some crucial cofactors (Rv0524, Rv1568, and Rv1569), of aminosugars (Rv3402c), of cell wall components (Rv0812), and in the metabolism of other molecules apparently important for bacterial virulence and adaptation (Rv2231c, Rv2589, and Rv3329).

The next largest functional group of enzymes in Table 1 is the PLP-dependent lyases or synthetases (Figure 2). Remarkably, many of these enzymes are apparently involved in the handling of sulfur-containing compounds, in particular, the amino acid cysteine (Figure 3). Indeed, the *Mtb* genome encodes three distinct enzymes (Rv2334, Rv0848, and Rv1336), all formally active as cysteine synthases, albeit with different substrates and metabolic roles.^70,71^ Rv2334 (CysK1) produces l-cysteine from sulfide (obtained via the sulfur assimilation pathway) and *O*-acetyl-l-serine (derived from the glycolytic intermediate 3-phosphoglycerate).^34,44,72^ Rv0848 (CysK2) acts most efficiently as a sulfocysteine synthase but can also synthesize l-cysteine using sulfide (like Rv2334) and *O*-phospho-l-serine.^24,71^ Finally, Rv1336 (CysM) uses *O*-phospho-l-serine and a small sulfur carrier protein termed CysO as substrates.^33,34^ Notably, l-cysteine can also be obtained from l-methionine via the reverse transsulfuration pathway, whose last step is catalyzed by Rv1079, a bifunctional cystathionine γ-lyase/γ-synthase^27^ (Figure 3).

**Figure 3:**
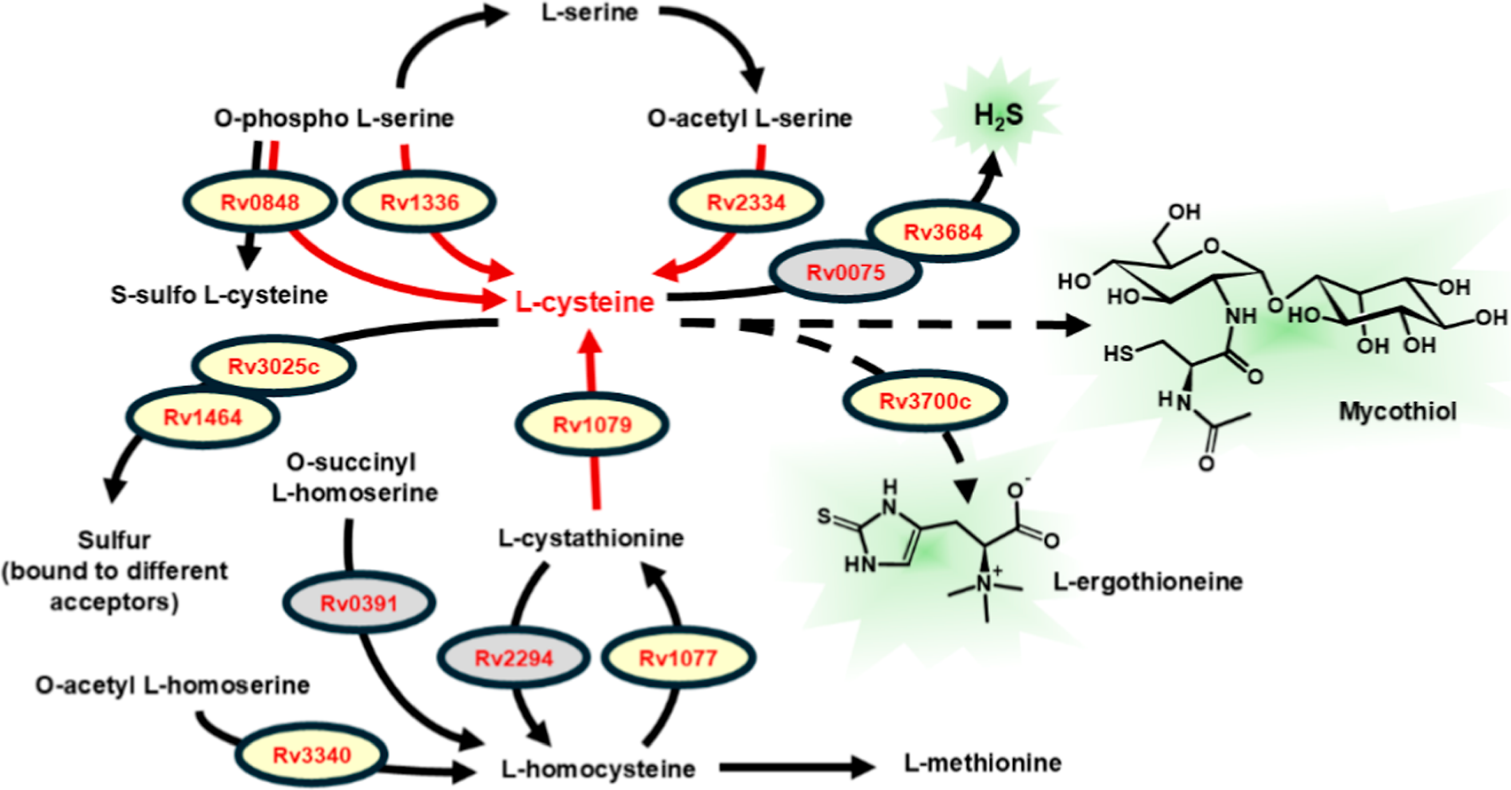
Metabolism of sulfurated amino acids and related thiols in *Mtb*, highlighting the central role of l-cysteine and the multiple reactions through which it can be formed (red arrows). PLP-dependent enzymes (synthases and lyases) involved in this metabolism are indicated by their corresponding gene locus; a gray background signals those enzymes whose function is only putative (see Table 1). While the metabolism of sulfurated compounds in *Mtb* contains several reversible processes, most of the reactions schematized in this figure are essentially irreversible (e.g., the reaction catalyzed by Rv3700c), and in any case, the arrows indicate the metabolic flow in the cell. For example, while Rv1079 can function in vitro both as a cystathionine γ-lyase (synthesizing l-cysteine from l-cystathionine) and as a cystathionine γ-synthase (utilizing l-cysteine and *O*-succinylhomoserine to yield l-cystathionine), only the lyase reaction presumably occurs in intact mycobacteria.^27^

Cysteine is not only necessary for protein synthesis but also serves as a precursor for ergothioneine (through the interrelated compound γ-glutamylcysteine) and mycothiol, which is the principal low-molecular-weight thiol in mycobacteria^73^ (Figure 3). These thiolic compounds play key roles in protecting the bacterium from oxidative stress and in maintaining redox balance.^74^

When pathogens infect macrophages, the newly formed phagosomes undergo a maturation process and fuse with lysosomes.^75^ The resulting phagolysosomes, in addition to being acidic and containing hydrolases and antimicrobial peptides, exploit systems for the generation of oxidative and nitrosative stress in order to enhance the elimination of the pathogens.^76–78^

Indeed, reactive oxygen species (ROS) are highly toxic to bacteria,[Fn fn2] as they can either directly destroy DNA, protein, and lipids or indirectly damage the nucleic acids via oxidation of the nucleotide pool.^79,80^ To counteract these phenomena, pathogens synthesize low-molecular-weight thiols that protect their vital biological processes and modulate physiology to ensure their fitness in the presence of elevated ROS levels.^81,82^ Ergothioneine, mycothiol, and γ-glutamylcysteine have been shown to interplay to protect *Mtb* against various cellular stresses.^83^ The enzyme Rv3700c (EgtE) is a PLP-dependent C–S lyase that catalyzes the last step of ergothioneine biosynthesis. It cleaves pyruvate and ammonia from the reaction product of EgtC to yield ergothioneine.^84,85^ However, Rv3700c is not essential for ergothioneine biosynthesis in *Mtb*, since the level of ergothioneine was not significantly altered in mutant deficient in EgtE.^53^ EgtE shares homology with at least two other C–S lyases encoded in the *Mtb* genome, namely, Rv3684 (Csd, 30% identity) and Rv3025c (IscS, 25.6% identity). It is possible that either or both of these enzymes may compensate for the loss of EgtE. Supporting this hypothesis, ergothioneine production in the Δ*iscS* H37Rv mutant was found to be lower relative to its parental wild-type strain.^45^ Alternatively, free PLP might induce the nonenzymatic synthesis of ergothioneine in the absence of EgtE, as previously shown in vitro.^86^

Generally speaking, the fact that the PLP-dependent enzymes involved in the transformations shown in Figure 3 are usually nonessential in vitro (Table 1), attests at least in part to the high degree of redundancy observed in this branch of metabolism (Figure 3) but also, possibly, to the relatively less demanding conditions that bacteria encounter in cell culture as compared to inside macrophages.

Other than aminotransferases and lyases/synthetase, the remaining functional groupings in Figure 2 are much smaller and include decarboxylases (four enzymes) and other enzymes involved in one-carbon metabolism, cofactor biosynthesis, and glycogen metabolism.

### “Unknown” PLP-Dependent Enzymes in *M. tuberculosis*

While for most entries in Table 1, the precise catalytic function can be considered established or very likely (based on a strong similarity to functionally validated enzymes), for other gene products the activity remains uncertain to various degrees.

One particular case is Rv2148c, i.e., the *Mtb* ortholog of a protein very conserved in bacteria and eukaryotes, known as YggS in *Escherichia coli* and as PLPBP (PLP binding protein) in humans. It is known that these Rv2148c homologues are involved in maintaining the homeostasis between different vitamers of vitamin B6 ^87^ but no clear catalytic function has been established for them.^87–89^ Nonetheless, a catalytic role seems likely, as these proteins structurally belong to the “Fold-type III” group of PLP-dependent enzymes (the same as alanine racemase and some decarboxylases; Supporting Information, Table S1), they bind PLP via a conserved (“catalytic”) lysine and their genes in bacteria cluster often with those encoding other enzymes.

A more TB-specific enzyme worth of functional characterization is Rv0032 (BioF2), which is only present in the *M. tuberculosis* complex but absent in other pathogenic, opportunistic pathogenic, or nonpathogenic species of mycobacterium.^90^ Rv0032 is genomically annotated as a putative 8-amino-7-oxononanoate synthase (an enzyme involved in biotin biosynthesis) but apparently does not possess that activity. In *M. smegmatis*, where the *bioF* gene is essential for growth in the absence of exogenous biotin, *bioF* deletion mutants were not complemented by the expression of *Mtb* Rv0032 (*bioF2*), whereas they were complemented by the expression of Rv1569 (*bioF1*).^91^ Sequence-wise, Rv0032 has the signatures of an α-oxoamine synthase and the most similar functionally validated enzymes are bacterial serine palmitoyl transferases^92^ (even though the mycobacterial protein possesses an extra N-terminal domain). Serine palmitoyl transferase catalyzes the synthesis of 3-dehydro-d-sphinganine, which is the initial step in the biosynthesis of sphingolipids. However, while sphingolipids are ubiquitous in eukaryotes their presence in bacteria is relatively limited^93^ and never described in mycobacteria,^94^ casting doubts about the actual role of Rv0032.

The Rv0075 protein could be a cysteine desulfidase (aka desulfhydrase), i.e., an enzyme that generates hydrogen sulfide from l-cysteine, given a 38% identity to the *aecD* gene product of *Corynebacterium glutamicum*.^95^ Rv2294, while distantly similar to cystathionine β-lyases, might also be a desulfidase. Indeed, whereas *Mtb* hosts a validated cysteine desulfidase, namely, Rv3684, disruption of the corresponding gene was found to reduce but not to eliminate H_2_S production.^52^ Understanding which enzymes are responsible for sulfide production is not simply of academic interest, as it has been shown that exogenous (host-derived) H_2_S targets the electron transport chain to increase respiration and ATP concentration, thereby stimulating *Mtb* growth.^96^ In other bacteria, H_2_S production has been attributed to the enzymatic activity of cystathionine β-synthase and cystathionine γ-lyase, and it has been proven that disrupting H_2_S-producing genes increases oxidative stress and antibiotic susceptibility.^97,98^ Genes for cystathionine β-synthase and cystathionine γ-lyase are present in the *Mtb* genome (Rv1077 and Rv1079, respectively; see Table 1 and Figure 3), although evidence for their activity in H_2_S production has only been demonstrated via specific inhibitors.^52^

Rv2531c is a probable amino acid decarboxylase, distantly similar in particular to arginine decarboxylases. Recently, employing cryoEM, it was determined that the Rv2531c protein forms a tetramer in two different conformations and undergoes a PLP-induced transition to a dimeric state, revealing oligomeric plasticity that may support mycobacterial resilience under stress conditions.^99^ The study, however, did not clarify the activity of this enzyme, whose gene is not essential for growth in vitro.

Even though the Rv3329 gene product is annotated in some databases as a *S*-adenosylmethionine: 8-amino-7-oxononanoate aminotransferase, its sequence is 44% identical to that of the (functionally validated) taurine-pyruvate transaminase from *Rhodococcus opacus*,^100^ which may suggest a similar function for Rv3329. There is no knowledge of taurine metabolism in *Mtb*, but the enzyme could theoretically act on the host taurine and be involved in the retrieval of sulfur from the host. Many studies have highlighted the importance of sulfur acquisition and metabolism for bacterial survival and tissue invasion.^101,102^

### Other Genes and Gene Products Related to PLP-Dependent Enzymes

Table 1 includes genes whose products are, with high likelihood, proper enzymes. However, sequence homology searches in the *Mtb* genome also revealed some other gene products that, despite showing some clear homology to authentic PLP-dependent enzymes, presumably do not retain an enzymatic function. These are summarized in Table 2.

**Table 2:** Non-Enzymic Mycobacterial Gene Products Phylogenetically Linked to PLP-Dependent Enzymes

Rv locus	gene name	derived from/related to	significance	ref
Rv1413 + Rv1414	-	β-hydroxy d-amino acid ammonia lyase	fragments of a single ancestral gene	-
Rv1503c + Rv1504c	-	dTDP-4-amino-4,6-dideoxygalactose aminotransferase	fragments of a single ancestral gene	-
Rv1519	-	dTDP-4-amino-4,6-dideoxy-d-glucose aminotransferase	partial duplication of Rv3402c?	-
Rv2322c + Rv2321c	*rocD1* + *rocD2*	ornithine aminotransferase	fragments of a single ancestral gene	^103^
Rv3778c	-	cysteine desulfurase	expressed gene, noncatalytic product	-

All the genes in the table are nonessential, and for most of them, the gene products are too short to be typical PLP-dependent enzymes. In three cases, couples of adjacent genes appear to be the remnant of a full-length gene that had undergone a mutation and inactivation. Thus, these genes are likely nonfunctional. The best-characterized case is that of the Rv2322c/Rv2321c couple: a work by Hampel et al. showed that, while the two genes are indeed derived from an ornithine aminotransferase gene (ArgD; conserved in *M. smegmatis* and in other Mycobacteriaceae), they do not contribute to growth on l-arginine.^103^

Similarly, Rv1503c and Rv1504 (apparently derived from a gene involved in aminosugar metabolism), besides being nonessential in in vitro studies, were found deleted in some clinical isolates of *Mtb*,^104^ lending further support to the possibility that they represent nonfunctional gene relics.

A distinct case is that of Rv3778c, which encodes a protein lacking the conserved lysine residue responsible for covalent PLP binding (Figure 1). Because of this, despite being distantly related to cysteine desulfurases, selenocysteine lyases, and other enzymes catalyzing beta-elimination reactions, the Rv3778c gene product is likely noncatalytic. The protein could, however, retain some function for the cell, presumably unrelated to catalysis.

### Targeting PLP-Dependent Enzymes for Antimycobacterial Therapy: Background and General Considerations

In principle, the development of drugs that inhibit PLP-dependent enzymes in *Mtb* may offer some relevant advantages. First, there is a consensus from different studies^54–57^ that at least one-quarter of these enzymes are essential for growth and survival of the pathogen in vitro (Table 1) and the number may be greater in vivo. For example, while Rv1079 and Rv1569 do not seem essential for the in vitro growth of *Mtb* H37Rv, the corresponding genes were reportedly needed for survival in primary murine macrophages.^105^ Overall, the essential roles played by many PLP-dependent enzymes make them attractive targets for developing novel antimycobacterial drugs.

Furthermore, as noted, there are substantial differences in the composition of the mycobacterial and human PLPome (Supporting Information, Table S1), and such differences could in principle be exploited to design inhibitors that are selective for the bacterial enzymes, minimizing the risk of toxicity to human cells.

However, the presence of common mechanistic features among PLP-dependent enzymes, as well as the conservation of some chemical and structural features in the active sites, means that cross-reactivity with human PLP-dependent enzymes may occur even when these enzymes have different functions and phylogenesis. A further issue is that *Mtb* exhibits a high degree of metabolic redundancy, meaning that inhibition of one enzyme may be mitigated or offset by the activation of alternative pathways.

An instructive example of the potential and pitfalls for drugs that target PLP-dependent enzymes is provided by the case of d-cycloserine (DCS), an antibiotic employed in antimycobacterial therapy for over 50 years and that is currently a second-line drug in cases of multidrug resistant TB. DCS is a well-known inhibitor of alanine racemase (Alr; Rv3423c),^106^ an enzyme absent in humans that in mycobacteria catalyzes the reversible conversion of l-alanine into d-alanine, an essential component of the peptidoglycan layer. The antibiotic reacts, via its exocyclic amino group, with the PLP cofactor of Rv3423c-Alr, forming an external aldimine that subsequently evolves into an isoxazole adduct, thereby blocking the enzyme activity (Figure 4A).^106^ DCS also inhibits the (non-PLP-dependent) enzyme d-alanine-d-alanine ligase, which is apparently the primary target of the drug.^107^ At any rate, the racemase and the ligase act in the same pathway so that DCS is particularly effective in disrupting peptidoglycan biosynthesis, impacting the structural integrity of the bacterial cell wall and hence the ability of *Mtb* to resist external stresses.

**Figure 4:**
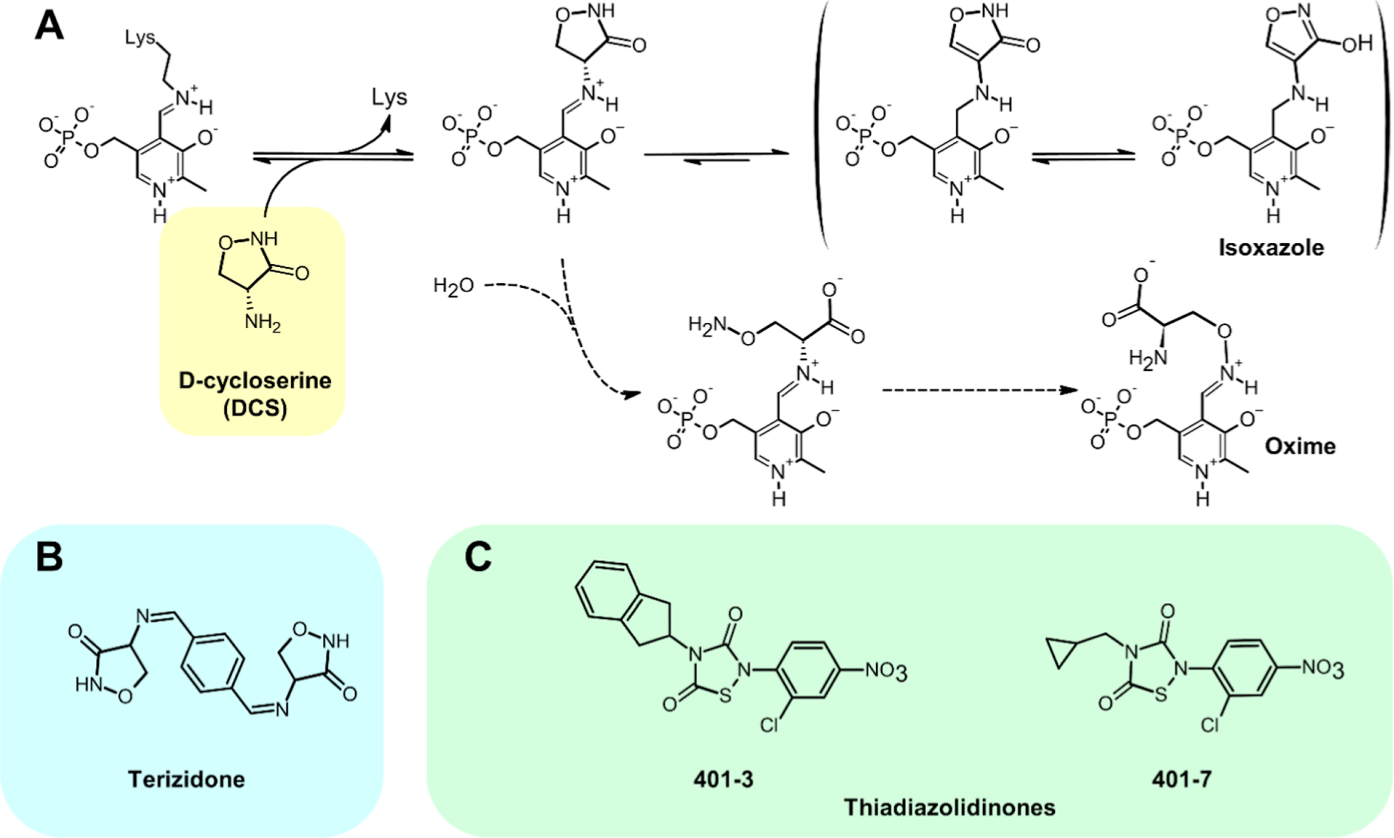
DCS and other drugs that target the mycobacterial Alr (Rv3423c). (A) The reaction of DCS with PLP passes through an internal aldimine intermediate and leads to the formation of some isoxazole adduct. The process is reversible, however, and over time the hydrolysis of the internal aldimine adduct can lead to a linearization of the cycloserine moiety, release of the linear adduct in solution (with its eventual rearrangement to a stable oxime) and reactivation of the enzyme.^106^ (B) Terizidone, in which two molecules of cycloserine are linked by one molecule of terephthalaldehyde. (C) Two thiadiazolidinone-based inhibitors of Alr, studied by Lee and co-workers.^108^ The shown denominations reflect the nomenclature used in the original paper.^108^

Although DCS is effective, its use is limited by neurotoxicity and other side effects.^109^ This may be related at least in part to the drug’s capacity to react with other PLP-dependent enzymes in addition to Alr^110,111^ and above all to its activity as an NMDA receptor agonist (off-target effects).^110^

Furthermore, contrary to previous assumptions, the mechanism of inhibition of alanine racemase by DCS is not properly irreversible, as the isoxazole adduct, while favored, remains in equilibrium with the internal aldimine (Figure 4A), which can undergo hydrolysis, leading to a slow reactivation of the enzyme. In essence, DCS should not be considered a purely irreversible inhibitor but rather a very slowly reacting substrate.^106,112^

Finally, while deletion of Rv3423c-*alr* prevents survival of the bacterium (Table 1), it has been shown that the production of d-alanine in *Mtb* can also proceed through a racemase-independent pathway involving the bifunctional enzyme Rv0812;^23,107^ this provides an example of metabolic redundancy, which in the long term might beget the development of drug resistance.

Similar problems have been encountered with other drugs designed to target alanine racemase. One of them is terizidone, a compound in clinical use (but not FDA-approved) that bears two cycloserine moieties (Figure 4B). Terizidone is in fact a prodrug that hydrolyzes in vivo to release cycloserine, thereby impairing peptidoglycan biosynthesis through Alr and d-alanine-d-alanine ligase inhibition. Its properties, limitations, and side effects are similar to those of plain DCS.^113,114^

Other medicinal chemistry efforts to target alanine racemase have focused on thiazolidinones,^108^ with the two best leads being shown in Figure 4C. Remarkably, their IC_50_ value toward Alr (Rv3423c) was found to be lower than 100 nM.^108^ However, their inhibition of *Mtb* growth was not uniquely associated with Alr inhibition, as the supply of exogenous d-alanine could not attenuate their effect.^108^

### Other Potentially Druggable PLP-Dependent Enzymes

Overall, besides DCS, there are only three FDA-approved drugs that target PLP-dependent enzymes:^115,116^ these are Carbidopa (an inhibitor of DOPA decarboxylase used in patients with Parkinson’s disease),^117^ Vigabatrin (an inhibitor of 4-aminobutyrate transaminase, used to treat epileptic seizures),^118^ and eflornithine (an ornithine decarboxylase inhibitor used mainly in the therapy of African trypanosomiasis).^119^ Notably, all these drugs act by binding via an amino or azido group to the carbonyl group of PLP, similar to the first enzymatic step depicted in Figure 1.^116^ This suggests two considerations. The first is that future inhibitors of other PLP-dependent enzymes probably would have to also react covalently with the carbonyl function of PLP, to ensure stable binding. The subsequent consideration, however, is that such inhibitors would also have the potential to react to some extent with PLP bound to enzymes other than the intended target, whereas covalent binding of the inhibitor to the cofactor may favor some reactivity that slowly destroys the inhibitor, as observed with DCS (Figure 4A).^106^

At any rate, inhibitors for PLP-dependent therapeutical targets are being actively investigated (e.g., inhibitors of ornithine transaminase, for the treatment of hepatocellular carcinoma^120^) and, in particular, there are ongoing drug discovery efforts targeting PLP-dependent enzymes in infectious diseases.^115,121,122^ As hinted in the previous section, ideal drug targets should be enzymes that are (a) essential for bacterial survival and (b) lack close functional and structural homologues in humans, to minimize off-target effects and reduce the risks of toxicity to human cells.

For example, among the enzymes in Table 1, Rv0524 (glutamate semialdehyde aminomutase) is an essential enzyme that catalyzes a reaction absent in mammalian metabolism, making it a potential target for the development of selective antimycobacterial agents, perhaps similar to the mechanism-based inhibitors previously generated.^123^

Other possible targets are enzymes that synthesize l-cysteine. As noted above, the *Mtb* genome encodes three l-cysteine synthases, namely, Rv2334 (CysK1), Rv0848 (CysK2), and Rv1336 (CysM), whereas humans do not possess these kinds of enzymes. This raises the possibility of developing antibiotics without a homologous target in the host. Given their role in cysteine production and stress resistance, cysteine synthases represent an attractive target.^70,124,125^ Schneider and co-workers identified several urea-based compounds that are able to target all three enzymes, which can potentially weaken the redox defense of *Mtb*,^124^ ultimately compromising survival during infection; however no in vivo tests have been reported as yet.

Other suggested targets have been Rv1612 (TrpB, tryptophan synthase),^126,127^ Rv1568 (BioA),^37,128–130^ and Rv3290c (Lat).^131^ These enzymes do not have counterparts in the human metabolism (Supporting Information, Table S1), and even if the genes corresponding to the last two were not essential for *Mtb* growth in vitro (Table 1), Rv3290c has been associated with mycobacterial persistence.^132,133^

In contrast to the above, Rv0884c (SerC) is essential and despite being isofunctional with the human phosphoserine aminotransferase, it has a substantial sequence divergence from it, with only 23.6% identity, making it an amenable new drug target.^134^

Indeed, even in the presence of a structural and/or functional human homologue (see Supporting Information, Table S1), designing inhibitors that selectively target the bacterial enzyme without affecting the host counterpart is a significant focus of current research. The subtle differences in the PLP-binding pocket and conformational dynamics may, in principle, allow the development of highly selective inhibitors. In this regard, it is worth noting that experimental structural information is available for about half of the enzymes in Table 1. The massive structural biology data that has been accumulated on the PLP enzymes of *Mtb* represents a particularly strong basis for the design of selective inhibitors: even considering that nowadays the three-dimensional structures of proteins can be predicted with good approximation from their sequences,^135,136^ actual experimental structures are still preferable for docking simulations.

Another possibility that has so far received little attention (and that is somewhat beyond the scope of this review) would be the inhibition of the PLP biosynthetic machinery, analogous to what has been proposed for other pathogens.^137^ In *Mtb*, biosynthesis of PLP from simple phosphorylated sugars is accomplished through a pathway that requires just two enzymes (Rv2606c and Rv2604c; Supporting Information, Figure S1).^13,138^ There is also a PLP salvage pathway, to which contributes, in particular, the enzyme pyridox­(am)­ine 5′-phosphate oxidase (PdxH; Rv2607).^138,139^ Nevertheless, as far as we know, there are no current investigations to develop inhibitors for the PLP biosynthetic enzymes of *Mtb*.

## Conclusions

PLP-dependent enzymes as a class play key roles in the metabolic network of *Mtb*. Their involvement in amino acid metabolism, cofactor synthesis, and stress response underscores their importance in the survival and pathogenicity of the bacterium. A large body of research has established the catalytic function of the vast majority of the enzymes in the mycobacterial PLPome, whereas a few still lack functional characterization. The knowledge accrued on these enzymes provides a peculiar view on mycobacterial metabolism. Moreover, the functional diversity observed within the mycobacterial PLP enzymes offers unique opportunities for therapeutic intervention.

However, despite some promises (and one drug already in use), several challenges remain in targeting PLP-dependent enzymes for TB treatment. One major concern is the conservation of the PLP reaction mechanisms, which raises the potential for cross-reactivity with human enzymes. Overcoming this requires some highly selective design strategy that exploits subtle differences in the enzyme structure and dynamics.

Furthermore, the redundancy of metabolic pathways in *Mtb* can sometimes allow the bacterium to compensate for the inhibition of a single enzyme. Under this respect, DCS is a fortunate case because the drug targets more than one enzyme.^107^ This suggests that a combination therapy, targeting multiple enzymes simultaneously, might be necessary to achieve a robust therapeutic effect and reduce the likelihood of resistance.^140^ In addition, synergy with standard antitubercular drugs may be warranted to achieve a bactericidal effect.

## Supplementary Material



## Footnotes

a For example, the small human redox protein mitoNEET is completely unrelated to classic PLP-dependent enzymes, yet it was recently reported to bind PLP and to show some l-cysteine transaminase activity in vitro,^141^ although it is not clear whether this reaction has any physiological relevance. The *Mtb* genome does not encode any obvious homologues of the human mitoNEET.

b Excess formation of ROS can also affect the lung cells, initiating a series of chemical reactions that result in the damage of cellular components, including proteins, lipids, and nucleic acids.^77^ To compensate for this burden, the lung has evolved numerous antioxidant defense mechanisms. Enzymatic antioxidant processes include superoxide dismutases (SOD), glutathione peroxidase, and catalase, whereas nonenzymatic processes involve molecules such as ferritin, ascorbic acid, and carotene.^77,78^

## Notes

Biosynthesis of vitamin B6 in *M. tuberculosis* and extended overview of the PLP-dependent enzymes encoded in the *M. tuberculosis* H37Rv genome (PDF)

## Abbreviations

PLP: pyridoxal-5′-phosphate
TB: tuberculosis
*Mtb*: *Mycobacterium tuberculosis*
EC: Enzyme commission
ROS: reactive oxygen species
DCS: d-cycloserine
