# Supplementary material for: The Pyridoxal-5′-Phosphate-Dependent Enzymes of Mycobacterium tuberculosis
Source: ACS Infect Dis. 2026 Jan 30;12(2):507–19. doi: 10.1021/acsinfecdis.5c00996 (PMC12910592; doi:10.1021/acsinfecdis.5c00996)
Supplement: Supplementary file 1 [file id5c00996_si_001.pdf]

Supplementary information to:

The pyridoxal-5'-phosphate dependent enzymes of *Mycobacterium tuberculosis*

Alessio Peracchi[1]*, Bienyameen Baker[2]

[1] Department of Chemistry, Life Sciences and Environmental Sustainability,
University of Parma, I-43124 Parma, Italy.

[2] SAMRC Centre for Tuberculosis Research; Division of Molecular Biology and Human Genetics, Faculty of Medicine and Health Sciences, Stellenbosch University, Cape Town, South Africa

* Corresponding author. Email: alessio.peracchi@unipr.it

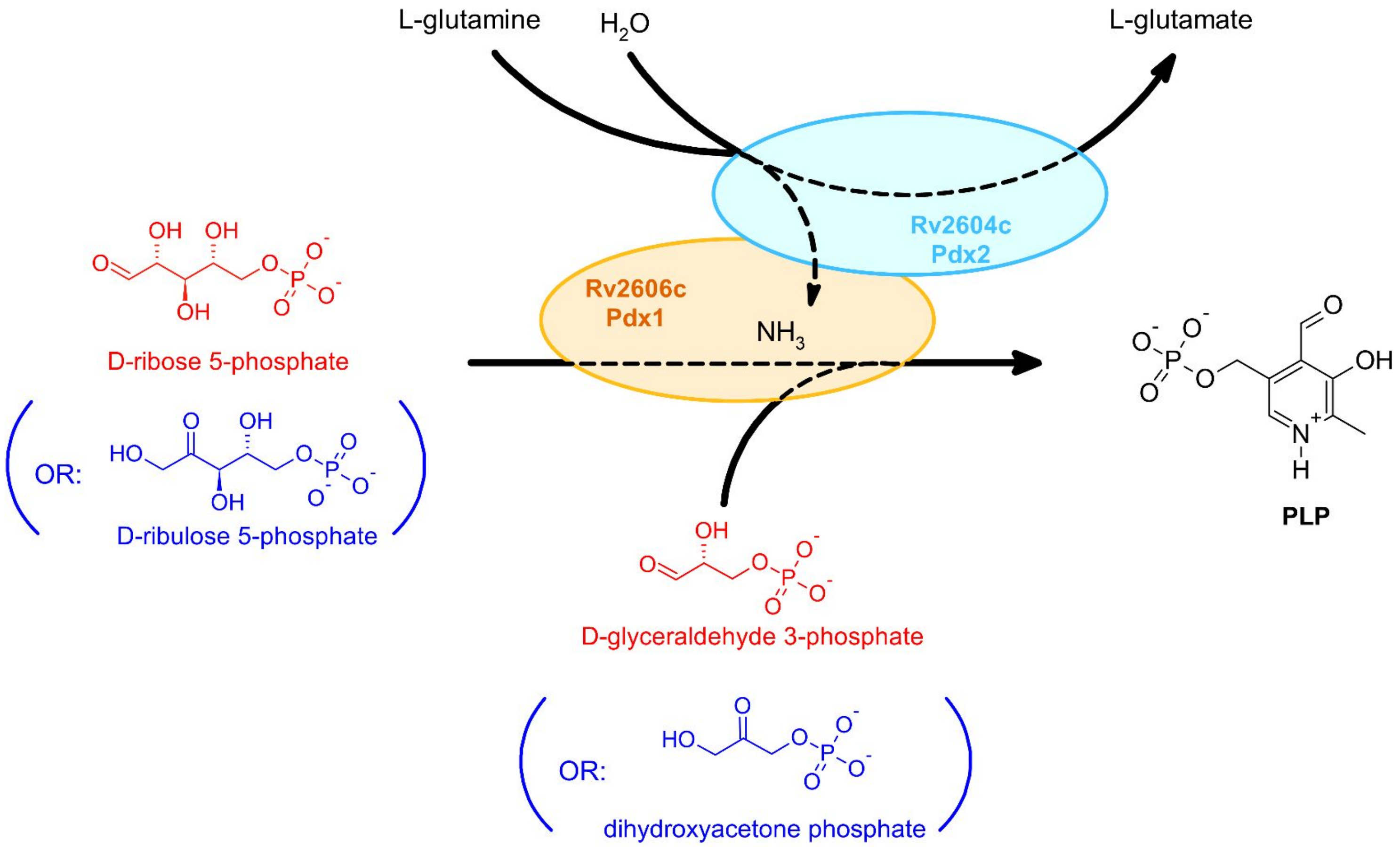

Supplementary figure 1 – Biosynthesis of vitamin B6 in *M. tuberculosis*. *Mtb* produces vitamin B6 via the so-called deoxyxylulose 5-phosphate-(DXP-) independent pathway [1], which is widespread among archaea, plants, fungi and a variety of bacteria (whereas a different, DXP-dependent pathway, which was extensively studied in *Escherichia coli*, is found in a smaller subset of bacteria)[2,3]. In this pathway, the biosynthesis of PLP from simple phosphorylated sugars is accomplished thanks to the activity of just two enzymes, termed Pdx1 and Pdx2 (Rv2606c and Rv2604c) [1] that together form a PLP synthase complex. The complex can employ either ribose 5-phosphate and glyceraldehyde-3-phosphate or ribulose 5-phosphate and dihydroxyacetone phosphate as carbon sources [1], whereas the PLP nitrogen atom derives from glutamine. The structure of Rv2606c (Pdx1) has been determined experimentally [4]. *Mtb* also possesses a salvage pathway for reconversion of pyridoxamine 5’-phosphate (or pyridoxine 5’-phosphate) into PLP, thanks to the FMN-dependent oxidase PdxH (Rv2607) [5,6].

Supplementary Table 1 – An extended overview of the PLP-dependent enzymes encoded in the *M. tuberculosis* H37Rv genome. This table extends and integrates Table 1 in the main text, by providing information about (1) the structural family ('fold-type') to which the enzyme belongs; (2) the NCBI refseqs corresponding to each gene product; (3) the occurrence of orthologs (>65% identical) of the *Mtb* genes in *Mycolicibacterium smegmatis*, which is commonly used as a nonpathogenic and fast-growing model organism to study mycobacteria; (4) the occurrence of structural homologs (arbitrarily defined as sequences showing >30% identity to the *Mtb* enzyme) encoded in the human genome and (5) the occurrence of functional homologs (that is, enzymes that are known to catalyze the same reaction) in the human metabolism, irrespective of their structure.

Gene locus	Gene name	Established or putative catalytic function	Validation	Ref.	Essential?	Fold type	NCBI refseq	Ortholog in *M. smegmatis* MC2 155	Homolog in *H. sapiens* Sequence	Function
Rv0032	*bioF2*	Serine C-palmitoyltransferase (EC 2.3.1.50) ?	-	-	1/4	I	NP_214546	-	GCAT	SPTLC2 ? SPTLC3 ?
Rv0070c	*glyA2*	Serine hydroxymethyltransferase (EC 2.1.2.1)	B	[7]	0/4	I	NP_214584	-	SHMT2	SHMT1, SHMT2
Rv0075	-	L-cysteine desulfidase (C-S lyase) (EC 4.4.1.28) ?	-	-	0/4	I	NP_214589	-	-	?
Rv0337c	*aspC*	Alanine aminotransferase (EC 2.6.1.2)	B	[8]	3/4	I	NP_214851	WP_003892107	GPT2	GPT, GPT2
Rv0391	*metZ*	O-succinylhomoserine sulfhydrylase (2.5.1.-)	G	[9]	1/4	I	NP_214905	WP_011727178	CTH	-
Rv0524	*hemL*	Glutamate-1-semialdehyde 2,1-aminomutase (EC 5.4.3.8)	-	-	4/4	I	NP_215038	WP_003892395	-	-
Rv0812	*pabC*	4-amino-4-deoxychorismate lyase / D-amino acid transaminase (EC 4.1.3.38/ 2.6.1.21) [g]	B	[10]	1/3	IV	YP_177757	WP_029104397	-	-
Rv0848	*cysK2*	S-sulfo-L-cysteine synthase (EC 2.8.5.1)	B	[11]	0/4	II	YP_177762	-	CBS	-
Rv0858c	*dapC*	Glutamine transaminase (EC 2.6.1.64/2.6.1.117)	-	-	0/4	I	NP_215373	WP_011730742	KYAT3	KIAT1, KYAT3
Rv0884c	*serC*	Phosphoserine aminotransferase (EC 2.6.1.52)	B	[12]	3/3	I	NP_215399	WP_003897096	-	PSAT1
Rv1077	*cysM2*	Cystathionine beta-synthase (EC 4.2.1.22)	B	[13]	0/4	II	YP_177782	WP_011730431	CBS	CBS
Rv1079	*metB*	Cystathionine gamma-lyase (EC 4.4.1.1)	B	[14,15]	0/3	I	NP_215595	WP_011730426	CTH	CTH
Rv1093	*glyA1*	Serine hydroxymethyltransferase (EC 2.1.2.1)	B	[7]	3/3	I	YP_177787	WP_011730415	SHMT2	SHMT1, SHMT2
Rv1178	-	Succinyldiaminopimelate transaminase (EC 2.6.1.17) [i]	B	[16]	0/4		NP_215694	WP_011730331	-	-
Rv1293	*lysA*	Diaminopimelate decarboxylase (EC 4.1.1.20)	B	[17,18]	4/4	III	NP_215809	WP_011730213	-	-

Rv1295	*thrC*	Threonine synthase (EC 4.2.3.1)	B	[19]	4/4	II	NP_215811	WP_011730211	-	-
Rv1328	*glgP*	Glycogen (glucan) phosphorylase (EC 2.4.1.1)	-		0/4	V	NP_215844	WP_011730188	-	PYGL, PYGM, PYGB
Rv1336	*cysM*	[CysO]-thiocarboxylate-dependent L-Cys synthase (EC 2.5.1.113)	B	[20,21]	0/4	II	NP_215852	WP_003896302	CBS	-
Rv1464	*csd*	Cysteine desulfurase, SufS-type (EC 2.8.1.7) [h]	B	[22][h]	4/4	I	NP_215980	WP_003894512	NFS1	NFS1
Rv1559	*ilvA*	Threonine ammonia-lyase (EC 4.3.1.19)	B	[23]	2/3	II	NP_216075	WP_003894570	SDS	SDS
Rv1568	*bioA*	S-adenosylmethionine : 8-amino-7-oxononanoate aminotransferase (EC 2.6.1.62)	B	[24]	1/4	I	NP_216084	WP_011728880	-	-
Rv1569	*bioF1*	8-amino-7-oxononanoate synthase (EC 2.3.1.47)	B	[25]	0/3	I	YP_177822	WP_011728881	GCAT	-
Rv1600	*hisC*	Histidinol-phosphate aminotransferase (EC 2.6.1.9)	B	[26]	4/4	I	YP_177823	WP_011728896	-	-
Rv1612	*trpB*	Tryptophan synthase beta subunit (EC 4.2.1.20)	B	[27]	3/3	II	NP_216128	WP_011728904	-	-
Rv1655	*argD*	Acetylornithine aminotransferase (EC 2.6.1.11)	B	[28]	4/4	I	NP_216171	WP_011729315	OAT	-
Rv1832	*gcvB*	Glycine dehydrogenase P protein (EC 1.4.4.2)	-	-	3/4	I	NP_216348	WP_011729226	GLDC	GLDC
Rv2148c	-	PLP-homeostasis protein (YggS family)	-	-	0/3	III	NP_216664	WP_014877975	PLPBP	PLPBP
Rv2210c	*ilvE*	Branched-chain amino acid aminotransferase (EC 2.6.1.42)	B	[29]	3/4	IV	NP_216726	WP_011729696	BCAT1	BCAT1, BCAT2
Rv2231c	*cobC*	Histidinol phosphate aminotransferase (EC 2.6.1.9) ?	B	[30]	1/3	I	NP_216747	-	-	-
Rv2294	-	Cystathionine beta-lyase / L-cysteine desulfidase (EC 4.4.1.8/4.4.1.28) ?	-	-	0/4	I	NP_216810	-	-	-
Rv2334	*cysK1*	O-acetylserine sulfhydrylase (EC 2.5.1.47)	B	[31]	0/3	II	YP_177868	-	-	-
Rv2531c	-	Putative amino acid decarboxylase (4.4.1.-) ?	-	-	0/4	I	YP_177889	-	-	-
Rv2589	*gabT*	4-aminobutyrate aminotransferase (EC 2.6.2.19)	-	-	0/4	I	NP_217105	WP_011728721[a]	OAT	ABAT
Rv3025c	*iscS*	Cysteine desulfurase, NifS-type (EC 2.8.1.7)	B	[32]	3/4	I	NP_217541	WP_011728292	NFS1	NFS1

Rv3290c	*lat*	L-lysine 6-transaminase (EC 2.6.1.36)	B	[33]	0/4	I	NP_217807	WP_003893146	ABAT	-
Rv3329	-	Taurine:pyruvate aminotransferase (EC 2.6.1.77)	-	-[f]	0/4	I	NP_217846	WP_003893069	PHYKPL	-
Rv3340	*metC*	O-acetylhomoserine sulfhydrylase (EC 2.5.1.49)	G	[34]	0/3	I	NP_217857	WP_003893059[b]	CTH	-
Rv3402c	-	dTDP-4-amino-4,6-dideoxy-D-glucose aminotransferase (EC 2.6.1.33)	B	[35]	0/4	I	NP_217919	-	-	-
Rv3423c	*alr*	Alanine racemase (EC 5.1.1.1)	B	[36]	4/4	III	NP_217940	WP_011727747	-	-
Rv3432c	*gadB*	Glutamate decarboxylase (EC 4.1.1.15)	B	[37]	0/4	I	NP_217949	WP_011727746		GAD1, GAD2
Rv3565	*aspB*	Valine:pyruvate aminotransferase (EC 2.6.1.66)	B	[8]	1/2	I	NP_218082	WP_014878528	KYAT1	-
Rv3684	*Cds1*	L-cysteine desulfhydrase (C-S lyase) (EC 4.4.1.28)	B	[38]	0/4	II	NP_218201	WP_011731115	CBS	-
Rv3700c	*egtE*	Hercynylcysteine S-oxide lyase (EC 4.4.1.36)	G	-	0/3	I	NP_218217	WP_011731155	-	-
Rv3722c	-	Aspartate aminotransferase (EC 2.6.1.1)	B	[8]	4/4	I	NP_218239	WP_011731178	-	GOT1, GOT2
Rv3772	*hisC2*	Aromatic amino acid transaminase (EC 2.6.1.57)	B	[26]	0/4	I	NP_218289	WP_011731224	-	TAT

[a] At least three close homologs exist in the *M. smegmatis* MC2 155 genome: in addition to WP_011728721, also WP_003892002 and WP_011731460), the latter of which shows a much lower sequence similarity to Rv2589 as compared to the other two.

[b] Two homologs exist in the *M. smegmatis* MC2 155 genome: WP_003893059 and WP_011726769, the second of which may have a distinct function based on its much lower similarity to Rv3340 and on its occurrence in a distinct genomic context.

References

1 Dick T, Manjunatha U, Kappes B & Gengenbacher M (2010) Vitamin B6 biosynthesis is essential for survival and virulence of *Mycobacterium tuberculosis*. *Mol Microbiol* 78, 980–988.

2 Fitzpatrick TB, Amrhein N, Kappes B, Macheroux P, Tews I & Raschle T (2007) Two independent routes of *de novo* vitamin B6 biosynthesis: not that different after all. *Biochemical Journal* 407, 1–13.

3 Denise R, Babor J, Gerlt JA & de Crécy-Lagard V (2023) Pyridoxal 5’-phosphate synthesis and salvage in Bacteria and Archaea: predicting pathway variant distributions and holes. *Microb Genom* 9, mgen000926.

4 Kim S & Kim K-J (2013) Crystal structure of Mycobacterium tuberculosis Rv2606c: A pyridoxal biosynthesis lyase. *Biochem Biophys Res Commun* 435, 255–259.

5 Ankisettypalli K, Cheng JJY, Baker EN & Bashiri G (2016) PdxH proteins of mycobacteria are typical members of the classical pyridoxine/pyridoxamine 5′-phosphate oxidase family. *FEBS Lett* 590, 453–460.

6 Pédelacq JD, Rho BS, Kim CY, Waldo GS, Lekin TP, Segelke BW, Rupp B, Hung LW, Kim S II & Terwilliger TC (2006) Crystal structure of a putative pyridoxine 5′-phosphate oxidase (Rv2607) from *Mycobacterium tuberculosis*. *Proteins: Structure, Function and Genetics* 62, 563–569.

7 Chaturvedi S & Bhakuni V (2003) Unusual structural, functional, and stability properties of serine hydroxymethyltransferase from *Mycobacterium tuberculosis*. *J Biol Chem* 278, 40793–40805.

8 Jansen RS, Mandyoli L, Hughes R, Wakabayashi S, Pinkham JT, Selbach B, Guinn KM, Rubin EJ, Sacchettini JC & Rhee KY (2020) Aspartate aminotransferase Rv3722c governs aspartate-dependent nitrogen metabolism in *Mycobacterium tuberculosis*. *Nat Commun* 11, 1960.

9 Nain VK, Barik V, Pandey M, Pareek M, Sharma T, Pal R, Tyagi S, Bajpai M, Dwivedi P, Panda BN, Kumar Y, Asthana S & Pandey AK (2025) A pH-dependent direct sulfhydrylation pathway is required for the pathogenesis of *Mycobacterium tuberculosis*. *Commun Biol* 8, 637.

10 Black KA, Duan L, Mandyoli L, Selbach BP, Xu W, Ehrt S, Sacchettini JC & Rhee KY (2021) Metabolic bifunctionality of Rv0812 couples folate and peptidoglycan biosynthesis in *Mycobacterium tuberculosis*. *J Exp Med* 218, e20191957.

11 Steiner EM, Böth D, Lössl P, Vilaplana F, Schnell R & Schneider G (2014) CysK2 from *Mycobacterium tuberculosis* is an *O*-phospho-L-serine-dependent *S*-sulfocysteine synthase. *J Bacteriol* 196, 3410–3420.

12 Bai G, Schaak DD, Smith EA & Mcdonough KA (2011) Dysregulation of serine biosynthesis contributes to the growth defect of a *Mycobacterium tuberculosis* crp mutant. *Mol Microbiol* 82, 180–198.

13 Bandyopadhyay P, Pramanick I, Biswas R, Sreedharan S, Singh S, Rajmani RS, Laxman S, Dutta S & Singh A (2022) S-Adenosylmethionine-responsive cystathionine β-synthase modulates sulfur metabolism and redox balance in *Mycobacterium tuberculosis*. *Sci Adv* 8, 97.

14 Wheeler PR, Coldham NG, Keating L, Gordon S V., Wooff EE, Parish T & Hewinson RG (2005) Functional demonstration of reverse transsulfuration in the *Mycobacterium tuberculosis* complex reveals that methionine is the preferred sulfur source for pathogenic mycobacteria. *J Biol Chem* 280, 8069–8078.

15 Saha B, Mukherjee S & Das AK (2009) Molecular characterization of *Mycobacterium tuberculosis* cystathionine gamma synthase-Apo- and holoforms. *Int J Biol Macromol* 44, 385–392.

16 Mandyoli L (2022) Annotating mycobacterial aminotransferases through function and structural studies; and Rv3208a is a small mycobacterial protein with a novel fold and quaternary structure. In *PhD dissertation* pp. 56–79. Texas A&M University, College Station, TX.

17 Gokulan K, Rupp B, Pavelka MS, Jacobs WR & Sacchettini JC (2003) Crystal structure of *Mycobacterium tuberculosis* diaminopimelate decarboxylase, an essential enzyme in bacterial lysine biosynthesis. *J Biol Chem* 278, 18588–18596.

18 Fogle EJ & Toney MD (2011) Analysis of catalytic determinants of diaminopimelate and ornithine decarboxylases using alternate substrates. *Biochim Biophys Acta* 1814, 1113–1119.

19 Covarrubias AS, Högbom M, Bergfors T, Carroll P, Mannerstedt K, Oscarson S, Parish T, Jones TA & Mowbray SL (2008) Structural, biochemical, and *in vivo* investigations of the threonine synthase from *Mycobacterium tuberculosis*. *J Mol Biol* 381, 622–633.

20 O'Leary SE, Jurgenson CT, Ealick SE & Begley TP (2008) *O*-phospho-L-serine and the thiocarboxylated sulfur carrier protein CysO-COSH are substrates for CysM, a cysteine synthase from *Mycobacterium tuberculosis*. *Biochemistry* 47, 11606–11615.

21 Ågren D, Schnell R, Oehlmann W, Singh M & Schneider G (2008) Cysteine synthase (CysM) of *Mycobacterium tuberculosis* is an *O*-phosphoserine sulfhydrylase: Evidence for an alternative cysteine biosynthesis pathway in *Mycobacteria*. *J Biol Chem* 283, 31567–31574.

22 Elchennawi I, Carpentier P, Caux C, Ponge M & Ollagnier de Choudens S (2023) Structural and biochemical characterization of *Mycobacterium tuberculosis* zinc SufU-SufS complex. *Biomolecules* 13, 732.

23 Sharma R, Keshari D, Singh KS & Singh SK (2017) Biochemical and functional characterization of MRA_1571 of *Mycobacterium tuberculosis* H37Ra and effect of its down-regulation on survival in macrophages. *Biochem Biophys Res Commun* 487, 892–897.

24 Mann S & Ploux O (2006) 7,8-Diaminoperlargonic acid aminotransferase from *Mycobacterium tuberculosis*, a potential therapeutic target: Characterization and inhibition studies. *FEBS J* 273, 4778–4789.

25 Bhor VM, Dev S, Vasanthakumar GR, Kumar P, Sinha S & Surolia A (2006) Broad substrate stereospecificity of the *Mycobacterium tuberculosis*7-keto-8-aminopelargonic acid synthase: Spectroscopic and kinetic studies. *J Biol Chem* 281, 25076–25088.

26 Nasir N, Anant A, Vyas R & Biswal BK (2016) Crystal structures of *Mycobacterium tuberculosis* HspAT and ArAT reveal structural basis of their distinct substrate specificities. *Sci Rep* 6, 18880.

27 Shen H, Yang Y, Wang F, Zhang Y, Ye N, Xu S & Wang H (2009) Characterization of the putative tryptophan synthase β-subunit from *Mycobacterium tuberculosis*. *Acta Biochim Biophys Sin (Shanghai)* 41, 379–388.

28 Nehvi IB, Quadir N, Khubaib M, Sheikh JA, Shariq M, Mohareer K, Banerjee S, Rahman SA, Ehtesham NZ & Hasnain SE (2022) ArgD of *Mycobacterium tuberculosis* is a functional N-acetylornithine aminotransferase with moonlighting function as an effective immune modulator. *Int J Med Microbiol* 312, 151544.

29 Venos ES, Knodel MH, Radford CL & Berger BJ (2004) Branched-chain amino acid aminotransferase and methionine formation in *Mycobacterium tuberculosis*. *BMC Microbiol* 4, 39.

30 Zarin S, Shariq M, Rastogi N, Ahuja Y, Manjunath P, Alam A, Hasnain SE & Ehtesham NZ (2024) Rv2231c, a unique histidinol phosphate aminotransferase from *Mycobacterium tuberculosis*, supports virulence by inhibiting host-directed defense. *Cell Mol Life Sci* 81, 203.

31 Schnell R, Oehlmann W, Singh M & Schneider G (2007) Structural insights into catalysis and inhibition of O-acetylserine sulfhydrylase from *Mycobacterium tuberculosis*: Crystal structures of the enzyme α-aminoacrylate intermediate and an enzyme-inhibitor complex. *J Biol Chem* 282, 23473–23481.

32 Rybniker J, Pojer F, Marienhagen J, Kolly GS, Chen JM, Van Gumpel E, Hartmann P & Cole ST (2014) The cysteine desulfurase IscS of *Mycobacterium tuberculosis* is involved in iron-sulfur cluster biogenesis and oxidative stress defence. *Biochem J* 459, 467–478.

33 Tripathi SM, Agarwal A & Ramachandran R (2015) Mutational analysis of *Mycobacterium tuberculosis* lysine ε-aminotransferase and inhibitor co-crystal structures, reveals distinct binding modes. *Biochem Biophys Res Commun* 463, 154–160.

34 Nzungize L, Ali MK, Wang X, Huang X, Yang W, Duan X, Yan S, Li C, Abdalla AE, Jeyakkumar P & Xie J (2019) *Mycobacterium tuberculosis metC* (Rv3340) derived hydrogen sulphide conferring bacteria stress survival. *J Drug Target* 27, 1004–1016.

35 Brown HA, Vinogradov E, Gilbert M & Holden HM (2018) The *Mycobacterium tuberculosis* complex has a pathway for the biosynthesis of 4-formamido-4,6-dideoxy-d-glucose. *Protein Science* 27, 1491–1497.

36 Strych U, Penland RL, Jimenez M, Krause KL & Benedik MJ (2001) Characterization of the alanine racemases from two Mycobacteria. *FEMS Microbiol Lett* 196, 93–98.

37 Rai R, Paroha R, Rai S, Singh AK, Chaurasia R, Agarwal N, Pandey MK & Chaurasiya SK (2025) Cloning, expression, purification, and characterization of glutamate decarboxylase (Rv3432c) from *Mycobacterium tuberculosis*. *Int Microbiol* 28, 1603–1616.

38 Kunota TTR, Rahman MA, Truebody BE, Mackenzie JS, Saini V, Lamprecht DA, Adamson JH, Sevalkar RR, Lancaster JR, Berney M, Glasgow JN & Steyn AJC (2021) *Mycobacterium tuberculosis* H2S functions as a sink to modulate central metabolism, bioenergetics, and drug susceptibility. *Antioxidants* 10, 1285.
